# Capsaicin: Effects on the Pathogenesis of Hepatocellular Carcinoma

**DOI:** 10.3390/molecules24132350

**Published:** 2019-06-26

**Authors:** Cristian Scheau, Ioana Anca Badarau, Constantin Caruntu, Gratiela Livia Mihai, Andreea Cristiana Didilescu, Carolina Constantin, Monica Neagu

**Affiliations:** 1 Department of Physiology, Carol Davila University of Medicine and Pharmacy, 050474 Bucharest, Romania; 2 Department of Dermatology, Prof. N.C. Paulescu National Institute of Diabetes, Nutrition and Metabolic Diseases, 011233 Bucharest, Romania; 3 Department of Embryology, Faculty of Dental Medicine, Carol Davila University of Medicine and Pharmacy, 050474 Bucharest, Romania; 4 Immunology Department, Victor Babes National Institute of Pathology, 050096 Bucharest, Romania; 5 Department of Pathology, Colentina University Hospital, 020125 Bucharest, Romania; 6 Faculty of Biology, University of Bucharest, 76201 Bucharest, Romania

**Keywords:** capsaicin, hepatocellular carcinoma, pathogenesis, regeneration, tumorigenesis, signaling pathways, oxidative stress, apoptosis, autophagy

## Abstract

Hepatocellular carcinoma (HCC) is one of the most frequent cancers, and to date, there have been very few drugs available that can improve survival, the most well-known being sorafenib. The pathogenesis of HCC is complex, involving multiple processes including abnormal cell and tissue regeneration, angiogenesis, genomic instability, cellular proliferation, and signaling pathway alterations. Capsaicin is a substance that holds increasingly high interest and is studied as a therapeutic option in a wide array of diseases. Several studies have investigated capsaicin roles in various stages of HCC oncogenesis. This paper aims to thoroughly detail the available information on the individual effects of capsaicin on the cellular mechanisms and pathways involved in HCC development, as well as investigate their possible cooperation and interferences. The synergistic antitumor effects of capsaicin and sorafenib are also addressed.

## 1. Introduction

Hepatocellular carcinoma (HCC) is the fifth most frequent cancer worldwide and the most common cause of death in cirrhotic patients, sometimes presenting atypical imaging or clinical features that can hinder its management [1,2]. The incidence of hepatocellular carcinoma is twice to four times higher in men than in women [3]. HCC yields an important economic burden, especially in societies with endemic infection with hepatitis B virus, such as the East Asian countries [4]. The pathogenesis of HCC is commonly considered as an overlap of long-lasting processes, such as hepatic cytolysis, inflammation, liver regeneration, and fibrosis, which ultimately favor the development of malignant foci [5].

Phytochemicals such as curcumin, resveratrol, oltipraz, and silibinin have been studied in the search for novel chemopreventive and chemotherapeutic agents for patients with hepatocellular carcinoma [6]. Dietary natural products have demonstrated antitumor properties in HCC, inhibiting angiogenesis, inducing apoptosis, suppressing cancer cell invasion and migration, and many more; such compounds include, but are not limited to: fruit (grapes and plums), vegetables (cruciferous, tomatoes, and asparagus) as well as spices (garlic and ginger) [7]. Flavanols have been demonstrated to decrease the risk of HCC onset, and the European Prospective Investigation into Cancer and Nutrition have recommended a high intake of substances in this class [8].

In recent years, capsaicin has captured the focus of attention as a novel agent in the diagnosis and treatment of a wide range of disorders [9]. Various papers cite apparently conflicting actions of capsaicin on tumorigenesis, as some reports identify it as a procarcinogenic substance, while others have demonstrated its anticarcinogenic effects [10]. The consumption of capsaicin reduces insulin resistance and is associated with a lower prevalence of obesity, suggesting its role in cell metabolism regulation [11,12]. Recent research shows that capsaicin metabolites can interfere with cell signaling pathways, thus inhibiting cellular differentiation and promoting carcinogenesis; therefore, approaching cellular metabolic reactions may be a new therapeutic strategy in cancer [13,14].

In vivo and in vitro studies have explored the antitumor roles of capsaicin in various cancers, such as lung, breast, gastric, and prostate cancers and cholangiocarcinoma [15]. Although it has been successfully applied clinically in dermatology and pain control, the usage of capsaicin in the treatment of cancers is limited [9,10].

A better understanding of the specific effects of capsaicin on the pathogenic mechanisms of HCC may reveal new directions in the treatment of this disease.

## 2. Capsaicin

**Figure 1:**
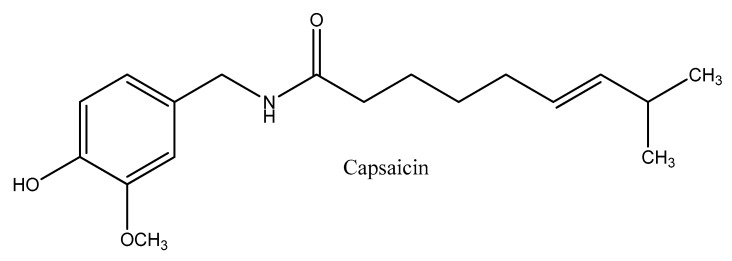
The chemical structure of capsaicin.

Capsaicin (*trans*-8-methyl-*N*-vanillyl-6-nonenamide) is a natural vanilloid, and the most abundant capsaicinoid in peppers, followed by dihydrocapsaicin (8-metil-*N*-vanillylnonanamide) [16] (Figure 1). Capsaicin is responsible for the pungency of chili peppers, and is biosynthesized through the condensation of products from the phenylpropanoid and the fatty acid pathways by capsaicin synthase [17]. It is located in the seeds and placental tissue of capsicum, and is an alkaloid with a melting point of 62–65 °C that is highly volatile, hydrophobic, odorless, and colorless. Structurally, capsaicin is made up of a central amide bond that connects a vanillyl head group to an aliphatic tail [18].

Capsaicin is an agonist for transient receptor potential cation channel subfamily V member 1 (TRPV1), which is a receptor that is activated by certain physical triggers, such as high temperatures (>43 °C) and acidic pH (<5.2), or biomolecules such as vanilloids or endogenous lipids. Other factors such as heat, acidic milieu, various mediators of inflammation, or different neurotransmitters are potential activators of TRPV1 [19,20,21,22,23,24,25,26,27,28,29,30,31,32,33].

When activated, the channel transiently opens and initiates depolarization, which is mainly due to the influx of Ca^2+^. As TRPV1 is commonly expressed in myelinated and some unmyelinated axons, depolarization upon capsaicin activation would send impulses to the spinal cord and brain, translating the effects of warming, tingling, itching, stinging, or burning. Since the capsaicin-sensitive nerve endings contain various neuropeptides, such as substance P (SP) and calcitonin gene-related peptide (CGRP), their activation is followed by a transient inflammatory process known as neurogenic inflammation, due to the local release of proinflammatory neuropeptides. Other factors, such as cytokines, prostaglandins, and mast cell activation products can also be involved neurogenic inflammation [34,35,36,37,38,39].

However, in case of repeated or prolonged capsaicin administration, after the initial phase of excitation, the sensory nerve fibers step into a more prolonged but reversible, refractory state of desensitization [40,41,42].

Moreover, repetitive capsaicin treatment reduces the neurogenic inflammatory reaction, probably by the depletion of neuropeptides from the sensory nerve endings [43,44]. Furthermore, when used in high concentrations or for a long period of time, capsaicin is associated with a series of cell metabolism alterations, including the suppression of mitochondrial respiration, inducing the progressive neurotoxic degeneration of cutaneous nerves and impairing nociceptor function for extended periods of time [45,46,47].

## 3. Hepatocellular Carcinoma

The pathogenesis of hepatocellular carcinoma (HCC) is a multistep process involving the progressive accumulation of genomic, transcriptomic, and epigenetic alterations pinpointing different molecular and cellular events [48]. Subsequently, certain cellular signaling pathways are involved in ensuring tumor survival and adapting to the microenvironmental changes arising in carcinogenesis [49] (Table 1).

The Wnt pathway acts through a yet unknown mechanism to promote stem cell renewal and maintain liver cancer stem cells properties; Notch expression may augment the effects of Wnt on stem cell self-renewal, and acts as a key regulator in the differentiation of stem cells into mature cell types. Alongside Notch and Wnt, Hedgehog acts through bone morphogenetic proteins (BMP) production signaling to promote differentiation, but also to limit intestinal stem cells niche (crypt) formation [62,63,64].

HCC cells are subjected to a hypoxic tumor milieu, where the main source of energy is represented by glycolysis induced by hypoxia-inducible factor (HIF)-1; the effect is further amplified in the case of transcatheter arterial embolization-pretreated HCC, where the energy necessary for cancer cell growth is obtained through HIF-1 mediated glycolysis [65]. The adaptation of tumor cells to hypoxia is possible with the help of transcription factor HIF-1α, which regulates several genes involved in tumor growth, including GLUT1. The suppression of GLUT1 and GLUT4 transcriptional activity is lost in mutant p53, which is commonly identified in tumor cell transformations, leading to an increase in the glucose requirements and metabolism of cancer cells [66].

The STAT3 signaling pathway can promote oncogenesis when stimulated by increased levels of proinflammatory cytokines, and appears to play an important role in the onset of HCC, as it is one of the initially affected pathways in this process. Sonic Hedgehog signaling, Ras/Raf/ mitogen-activated protein kinase (MAPK) signaling, the Notch pathway, phosphatidylinositol-3-kinase (PI3-K)/protein kinase B (Akt)/mammalian target of rapamycin (mTOR) and TGF-β signaling are other pathways that are also involved in HCC development. The configuration of diverse cell surface markers demonstrate that the alteration of these mentioned pathways is responsible for tumor growth through the facilitation of cancer stem cells development [49,67]. 

**Figure 2:**
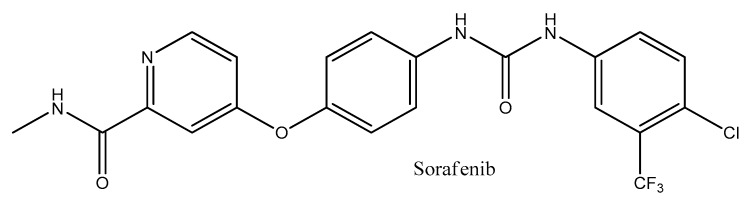
The chemical structure of Sorafenib.

Sorafenib is a multikinase (tyrosine kinase, angiogenesis, vascular endothelial growth factor) inhibitor used for the treatment of advanced HCC, with moderate results in term of prolonging life expectancy due to drug resistance development, and to date, it is the only molecular drug approved by the United States Food and Drug Administration [49] (Figure 2). A better understanding of the ways in which capsaicin interacts with the mechanisms of HCC pathogenesis may unveil new therapeutic options.

## 4. Current Roles of Capsaicin in the Treatment of Hepatocellular Carcinoma

In vivo and in vitro studies have uncovered a series of cellular mechanisms in HCC cells influenced by capsaicin. Some of the data are controversial, as there is little information available on this topic. The main findings are detailed below.

### 4.1. Specific Capsaicin Effects on the TRPV1 Receptor

Capsaicin exerts its effects on TRPV1, inducing an influx of calcium ions, and less importantly, sodium ions, and causing cellular activation [68]. Some studies implied that other mechanisms are also involved in translating the antitumor effects of capsaicin, as they were unable to demonstrate TRPV1 expression in HCC cells [69]. The concentrations of capsaicin that activate TRPV1 in normal conditions are far lower than the levels required to elicit anticarcinogenic actions, suggesting that its effects on inducing apoptosis or limiting cell proliferation are also facilitated by other pathways [69,70]. However, it is currently considered that TRPV1 is one of the main performers in carrying out the antitumor actions of capsaicin [67].

It is noteworthy that capsaicin binding to the TRPV1 receptor may be increased using a static magnetic field (SMF), thus enhancing the anti-cancer effect of capsaicin on HepG2 (human hepatoblastoma cell line) cells through caspase-3 apoptosis [71].

Diversely, the activation of TRPV1 by dietary capsaicin may prevent non-alcoholic fatty liver disease by eliciting anti-inflammatory roles on the liver cells of wild-type mice, and also activating peroxisome proliferator-activated receptor (PPARδ), which stimulates the expression of light chain 3B (LC3-II) and Beclin1, which induce autophagy in HepG2 cells [72]. Non-alcoholic fatty liver disease was identified—alongside chronic liver viral infection and ethanol abuse—as a cause of HCC in several studies, by recognizing significant associations between HCC developed on a non-cirrhotic liver and various metabolic disorders, including obesity and type 2 diabetes mellitus [73]. No relevant clinical studies were found pertaining to the role of capsaicin on other precursor conditions of HCC, such as viral hepatitis B and C, or liver cirrhosis.

### 4.2. Effects on Tumor Differentiation

The hedgehog (HH) pathway is critical in the embryonic development of the liver due to the regulation of cell differentiation and proliferation and the control of the expression of epithelial markers on liver cells. However, HH may be reactivated in HCC, and the effects on molecular signaling and cell proliferation can lead to significant cancer progression and metastasis [74].

In HCC cells, autophagy can be prevented through HH agonists that stimulate HH signaling, and it may be induced by GANT61, which inhibits GLI1/2 and suppresses HH [75]. Data regarding the effects of capsaicin on HH signaling in the HCC pathogenesis is scarce. Capsaicin demonstrates significant antiproliferative activity in cholangiocarcinoma by restricting the activation of the HH pathway and limiting its effects on tumorigenesis, on in vivo and in vitro studies [76]. On the other hand, in bladder cancer, capsaicin triggered autophagic cell survival and stimulated tumor invasion through epithelial–mesenchymal transition (EMT) while promoting chemoresistance through the regulation of the HH pathway [77].

### 4.3. Effects on Genomic Stability and Cell Cycle Regulation

In conditions of cellular stress or cell damages, cell-cycle arrest can be triggered at different checkpoints through the action of p53, which is a central cell-cycle regulator protein. In order to exert its actions, p53 needs to undergo some posttranslational modifications that are responsible for its activated form [78]. AMP-activated protein kinase (AMPK) plays a major role in tumor development due to its ability to induce a p53 mediated cell-cycle arrest, which is an action that is meant to facilitate cell survival in periods of metabolic stresses appearing at different times and threatening the normal cell homeostasis [79].

Metabolic cell homeostasis is governed by the key energy sensor AMPK, so new therapeutic options are explored by targeting AMPK in order to control carcinogenesis [80]. Capsaicin activates AMPK in HepG2 cells via the TRPV1 receptor, which is an action that relies on intracellular calcium and calcium/calmodulin-dependent protein kinase beta (CaMKKβ) [13]. Capsaicin-activated AMPK in turn phosphorylates proteins involved in cell-cycle regulation and apoptosis, such as p21 and p53, as well as proteins involved in autophagy, thus acting as a tumor suppressor and playing a central role in a series of intracellular signaling pathways [81].

AMPK activation in HepG2 cells can be reduced through the use of intracellular calcium chelators, or by blocking TRPV1 with capsazepine, demonstrating that both intracellular calcium and TRPV1 are key limiting factors in the activation of AMPK [13]. On the other hand, it was shown that activating TRPV1 channels with capsaicin can accelerate HepG2 cells’ migration through an intracellular Ca^2+^ influx mechanism [82].

Cytochrome c is an important regulating factor in the generation of mitochondrial membrane potential. Cytochrome c is released to the cytosol through the permeabilization of the mitochondrial outer membrane, which is triggered by the swelling of the mitochondrial matrix: a process that is regulated by the Bcl-2 family proteins [83,84,85].

Capsaicin significantly decreases both cytosolic p53 protein and mitochondrial-released cytochrome c concentrations in HepG2 cells treated with BAPTA, leading to a reduction in reactive oxygen species (ROS) production with subsequent decrease in cell DNA damage. These facts indicate that the key regulator in this process is intracellular Ca^2+^, which intervenes in the triggering of apoptosis in HCC cells treated with capsaicin. Capsaicin causes cytochrome c release from the mitochondria, as demonstrated on analytic cellular studies, and also modulates the activity of the B-cell lymphoma 2 (Bcl-2) family proteins; both these effects seem to be dose-dependent, which are observations that were validated in studies with variable capsaicin concentrations [86].

### 4.4. Effects on Cell Proliferation and Survival

The epidermal growth factor (EGF)–epidermal growth factor receptor (EGFR) pathway is regarded as a potential target for the treatment of HCC, due to its apparent involvement in carcinogenesis, as well as inflammation, which is currently considered a bridge to HCC development. EGF is responsible for modulating key cellular functions that enhance tumor progression in HCC cells, such as regeneration, proliferation, and DNA synthesis [87]. HCC development is significantly reduced when EGFR activity is hindered by gefitinib, which is a selective inhibitor with a demonstrated anticarcinogetic role in HCC from in vivo studies [88]. As previously stated, the prolonged administration of capsaicin or in high concentrations reduces the neurogenic inflammatory response. In an in vivo study, capsaicin injections prevented the secretion of SP from sensory nerve fibers, also affecting the expression of EGF/EGFR in granulation tissues [89]. There is hope that a similar type of response in hepatic tissue can reduce the risk for the development of HCC or may contribute to the antitumoral effect of capsaicin.

The phosphatidylinositol-3-kinase (PI3K)/Akt pathway favors cell survival in metabolic stress conditions, and can inactivate pro-apoptotic factors as well as activate a positive regulator of the survival factor NFκB [90]. The mammalian target of rapamycin (mTOR) kinase is involved in multiple essential cellular processes, including cell proliferation, cell survival, and autophagy. Most effectively studied during starvation, mTOR inhibits autophagy; conversely, mTOR inhibition can induce autophagy, activating autophagosomes that engulf cellular components and participate in the formation of autolysosomes, which will degrade the enveloped products [91]. Even though the PI3K/Akt and the mTOR pathways involve distinct signaling molecules, they are often considered as a singular pathway due to their interconnections and common roles in influencing key cellular functions [90]. When activated, the PI3K/Akt/mTOR signaling pathway stimulates cell proliferation with an overall increase in tumor growth, invasiveness, and chemoresistance by causing an imbalance in cellular development homeostasis [90]. Capsaicin may inhibit the phosphorylation of Akt, as well as that of mTOR. The inhibition of the Akt pathway suppresses autophagy through mTOR downstream involvement, while the direct inhibition of the mTOR pathway can lead to the induction of autophagy on the NPC-TW01 nasopharyngeal carcinoma cell line; in summary, capsaicin can regulate autophagy by inhibiting the Akt/mTOR signaling pathway [92]. 

Capsaicin can upregulate the activity of the signal transducer and activator of transcription 3 (p-STAT3), inducing autophagy in HepG2 cells by triggering the generation of ROS. When autophagy is inhibited, capsaicin induces apoptosis in studies on the same HCC line [93]. Conversely, a study on melanoma cells suggested that STAT3 signaling may be essential to the onset of metastasis through the increase of the expression of MMP-2, enhancing tumor invasiveness and metastasis rates by favoring the EMT of tumor cells [94].

Another study has shown that capsaicin may exhibit a chemopreventive role by inhibiting the growth of SK-Hep-1 hepatocellular carcinoma cells in a dose-dependent manner through the induction of apoptosis mediated by a caspase-3-dependent mechanism [95].

Moreover, capsaicin may induce apoptosis through endoplasmic reticulum (ER) stress and the subsequent ER release of Ca^2+^, as demonstrated by the induced rise in cytoplasmic GADD153 levels, which lead to GRP78 nuclear translocation [86]. Furthermore, capsaicin induces apoptosis in HepG2 cells by reducing the levels of xIAP and cIAP1 proteins, which are inhibitors of caspase-3 activation [86].

Other studies have suggested that capsaicin may induce apoptosis in the HepG2 cells by activating a phospholipase C (PLC)-dependent intracellular Ca^2+^ release pathway, as demonstrated by a significant suppression of capsaicin effects when intracellular Ca^2+^ release and PLC are blocked through specific inhibitors. In contrast, extracellular Ca^2+^ chelation with EGTA does not significantly interfere with capsaicin-induced apoptosis and the rise of intracellular Ca^2+^ [96].

Capsaicin increases the expression of tumor necrosis factor-related apoptosis-inducing ligand (TRAIL) receptor DR5 through a Ca^2+^ influx-dependent activation of SP1, inducing apoptosis in HCC cells that present some degree of resistance to TRAIL-mediated apoptosis [97].

### 4.5. Effects on Tumor Angiogenesis

Angiogenesis is essential for tumor progression, and is defined as the process of creating new blood vessels through the proliferation and differentiation of endothelial cells in the conditions of a degraded extracellular matrix, and under the influence of numerous promoting factors. Vascular endothelial growth factor (VEGF) has been intensively studied due to its main role in angiogenesis, where it acts as an important regulator, alongside several other growth factors and cytokines [70].

VEGF is produced in HCC cells in concentrations that are correlated with tumor size and disease stage. Additionally, the vascular endothelial growth factor receptor (VEGFR) is also expressed in HCC cells, suggesting that VEGF/VEGFR signaling is involved in angiogenesis and subsequent tumor progression in a paracrine, as well as an autocrine pathway [98].

Capsaicin is able to suppress VEGF-mediated angiogenesis on a cellular level even at very low concentrations, by significantly diminishing the effects on the proliferation and differentiation of endothelial cells. The anti-angiogenic dose-dependent effects of capsaicin consist of inhibiting the endothelial cell proliferation induced by VEGF, as well as by fibroblast growth factor [99]. 

Literature data regarding the effects of capsaicin on VEGF signaling in HCC cells is scarce, but in vitro and in vivo inhibition of the growth of HCC cells was demonstrated using anti-VEGF monoclonal antibodies [98]. As the inhibition of VEGF decreases the proliferation of HCC cells, capsaicin may also act as an anti-angiogenic agent.

### 4.6. Effects on Oxidative Stress

ROS are increased in cancer cells, in effect to the particularities of the tumor cell metabolism. ROS can facilitate tumor progression in HepG2 cells, amplifying the rates of invasion and metastasis, but these effects can be repelled through the use of antioxidants. As such, an adequate control of ROS formation can consequently lead to a restriction of HCC aggressiveness, providing a better control in the progression of the disease [100].

Oxidative stress may also play an important role in preventing tumor progression through apoptosis. It was suggested that capsaicin can induce apoptosis in HepG2 cells through a NADPH oxidase-mediated generation of ROS, which is supported by the observation that using specific NADPH oxidase inhibitors leads to a suppression of the ROS generation and apoptosis induced by capsaicin [101]. 

Capsaicin-induced ROS generation can lead to membrane sphingophospholipids hydrolysis, with the subsequent release and accumulation of phosphocholine as well as, more importantly, ceramide [102]. Ceramide accumulation can determine the activation of TRAIL as well as several other pro-apoptotic genes, leading to cell metabolism impairment and apoptosis [103]. 

Capsaicin can inhibit the activity of tumor-associated NADH oxidase (tNOX) by suppressing the expression of domain transcription factor POU3F2, restricting tumoral growth and inducing apoptosis [104]. In order for the anticarcinogenic effects of capsaicin to be carried out, tNOX expression on the cell surface is essential [105].

Despite these observations, capsaicin-induced metastasis was detected in an animal model of colorectal cancer, and the mechanism was demonstrated to involve ROS modulation by low concentrations of capsaicin [106]. On the other hand, an excess of capsaicin is cytotoxic on HepG2 cells, and normal hepatocytes to a smaller extent, by collapse of the mitochondrial membrane potential with ROS formation [107]. This proves that capsaicin may have opposite dose-dependent effects via the same pathways, and further investigation is required into establishing the definitive effects of capsaicin and the specific thresholds of activity for the desired effects.

### 4.7. Capsaicin and Sorafenib Synergistic Antitumor Effects

Sorafenib is a small molecule chemotherapeutic drug that has been approved for the treatment of several cancers, acting as an apoptosis inducer and inhibitor of tumor cell proliferation and angiogenesis. Acting as a multikinase inhibitor, sorafenib blocks the activity of multiple targets, which are present either in the tumor cell (such as CRAF, BRAF and c-KIT) or the adjacent neoangiogenesis vessels (CRAF, VEGFRs, and platelet-derived growth factor receptor β) [108,109,110]. One of the yet unsurmounted downsides of sorafenib is the development of drug resistance, modulated by the crosstalking PI3K/Akt and the mitogen-activated protein kinase (MAPK)/extracellular signaling-regulated kinase (ERK) pathways [111]. Sorafenib does not directly block the PI3K/Akt pathway, but the inhibition of this pathway elicits strong antitumoral effects that increase the efficacy of sorafenib [112]. The effects of sorafenib are compromised in sustained exposure due to the direct or mTOR-mediated activation of Akt, which is a phenomenon that occurs in sorafenib-resistant cells [111]. The MAPK/ERK pathway is involved in the development of HCC, and consists of numerous signaling proteins that may be activated by the effect of various stimuli on cell surface receptors [113].

In a recent study, the combination of capsaicin and sorafenib demonstrated significant anticarcinogenic properties on LM3 HCC cells, restricting tumor cell growth, invasion, migration, and inducing apoptosis and autophagy, both in vitro and in vivo. The combination treatment inhibited the PI3K/Akt/mTOR pathway and decreased EGFR, Bcl-2, and p62 concentrations, while increasing the cytosol levels of various signaling proteins (such as caspase-3, Bax, beclin-1) and autophagosome proteins LC3A/B-II. Also, the co-treatment demonstrates antimetastatic effects by inhibiting EMT mediators such as MMP-2 and MMP-9, as well as mesenchymal markers N-cadherin and vimentin, while increasing the levels of adhesion molecule E-cadherin [114].

The combination of capsaicin and sorafenib has demonstrated superior antitumoral effects over singular treatment with either drug, in HepG2 as well as Huh-7 hepatocellular carcinoma cell lines [81]. The co-treatment was also a strong inducer of apoptosis in PLC/PRF/5 hepatoma cells, through an increase in Bax expression and corresponding decrease in Bcl-2 expression [69]. The two agents also demonstrate strong combined antitumoral activity by the co-suppression of signal transducer and activator of transcription 3 (STAT3), leading to a restriction in the proliferation of HCC cells [69].

A recent study reported that DE605, a novel c-Met inhibitor, activates the MAPK/ERK pathway, while sorafenib inhibits it, leading to a combined antitumor effect in HCC cells [115]. In a similar way, the increased level of phosphorylated ERK induced by capsaicin is subsequently decreased by high concentrations of sorafenib, which demonstrates how the synergistic antitumor effects are achieved [69].

Capsaicin inhibits the PI3K/Akt/mTOR pathway in combination with sorafenib, playing an important role in potentially overcoming the development of drug resistance caused by sorafenib-induced PI3K/Akt activation [81].

## 5. Discussion

**Figure 3:**
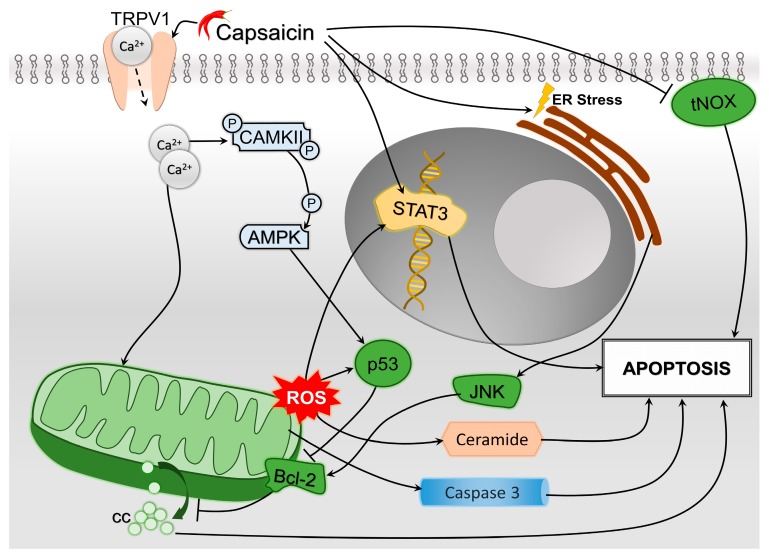
Synthetic depiction of the involvement of capsaicin in the mechanisms of hepatocellular carcinoma (HCC) cells’ apoptosis. Major pathways include endoplasmic reticulum (ER) stress, with subsequent activation of B-cell lymphoma 2 (Bcl-2) proteins through c-Jun N-terminal kinase (JNK) pathway, as well as mitochondrial reactive oxygen species (ROS) formation due to calcium inflow, which determines ceramide accumulation. Also, apoptosis is achieved by the inhibition of tumor-associated NOX (tNOX) and promoting the signal transducer and activator of transcription 3 (STAT3) expression, as well as mitochondrial caspase-3 release. Increased Bcl-2 levels will decrease cytochrome c (cc) concentrations, preventing apoptosis.

**Figure 4:**
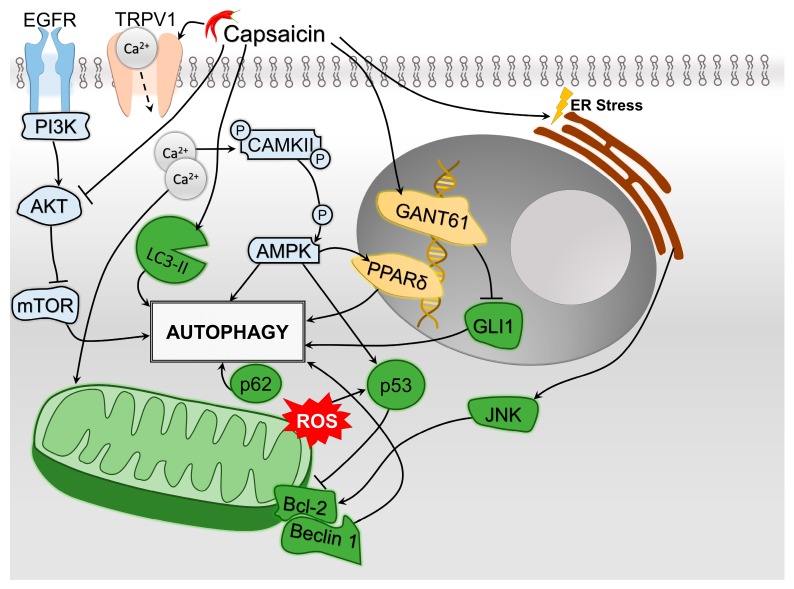
Synthetic depiction of the involvement of capsaicin in the mechanisms of HCC cells’ autophagy. Major pathways include endoplasmic reticulum (ER) stress, with subsequent activation of B-cell lymphoma 2 (Bcl-2) proteins through c-Jun N-terminal kinase (JNK) pathway, as well as the modulation of epidermal growth factor receptor (EGFR)/phosphoinositide 3-kinases (PI3K)/protein kinase B (AKT)/mechanistic target of rapamycin (mTOR) pathway. Also, autophagy may be achieved through autophagosome stimulation through the lipid modified form of microtubule-associated proteins 1A/1B light chain 3B (LC3-II) stimulation and promoting peroxisome proliferator-activated receptor gamma (PPAR-δ) and GANT61 expression. Mitochondrial reactive oxygen species (ROS) formation due to calcium inflow will induce an increase of p53, which subsequently will target the Bcl-2/Beclin 1 pathway, leading to autophagy. The accumulation of p62 may also lead to autophagy.

All major pathways of HCC cells oncogenesis and tumor progression may be affected by capsaicin to some extent. In most cited studies, capsaicin promotes apoptosis and autophagy and decreases cellular proliferation and angiogenesis. Nevertheless, interference was observed between the apoptosis and autophagy mechanisms. In this paper, we identified the following pathways through which capsaicin modulates the triggering of apoptosis: caspase-3 activation, ROS generation with direct effects, or indirect via STAT3 upregulation and ceramide accumulation, and inhibition of tNOX (Figure 3). Capsaicin induces autophagy by: stimulating LC3-II conversion and p62 accumulation, blocking the Akt/mTOR pathway, activating AMPK (directly and possibly via PPARδ), inhibiting GLI1 in the HH pathway as well as stimulating the Bcl-2/Beclin 1 pathway by inducing ER stress (Figure 4). Interestingly, while Bcl-2 acts as a waypoint for the autophagy pathway in HCC cells, it also blocks cytochrome c release from the mitochondria, disabling its role in signaling apoptosis. The Bcl-2 family regulates apoptosis by balancing the activation of its pro-apoptotic and antiapoptotic members [116]. Capsaicin may play a role in tipping the scale between the two sides; by inhibiting apoptosis, it in fact promotes autophagy. A further hurdle in establishing the clear role of capsaicin in the tumoral metabolism is that the ROS generated by capsaicin subsequently induce an increase in p53, which facilitates the outer mitochondrial membrane by interacting with Bcl-2, leading to a cytosolic release of cytochrome c that triggers apoptosis [117]. At the same time, capsaicin-induced ER stress will activate the c-Jun N-terminal kinase (JNK)/Bcl-2 pathway, which decreases the mitochondrial outer membrane permeability and inhibits the release of cytochrome c from the mitochondria [118]. 

The combination of capsaicin and sorafenib seems promising in the treatment of HCC, as the two substances act concurrently on inducing apoptosis and preventing cellular proliferation, while capsaicin also prevents the development of resistance to sorafenib by targeting the PI3K/Akt pathway [114].

Another recent potential therapeutic synergy might be to enhance the antitumor effects of capsaicin through the use of SMF, which causes a conformational change in the TRPV1 ion channel, further stimulating the downstream capsaicin-induced signaling pathways [71].

Despite the mainly positive effects of capsaicin on HCC that have been observed in various in vitro (Table 2) and in vivo (Table 3) studies, there are also studies suggesting a pro-carcinogenic effect of capsaicin [119]. The dual role of capsaicin on cancer cells was demonstrated on various phenotypes, which are mainly epidermal and adenocarcinomas, and seems to be dose-dependent [106]. Nevertheless, the complexity and overlapping of intracellular pathways and the intricate balance of factors involved obscure the definitive role of capsaicin on HCC and suggest that further studies are required in order to validate these findings.

Capsaicin remains one of the most promising candidates for the treatment of a wide array of diseases, with potential applications in the management of pain, inflammation, and several types of cancers [7,9,10,15,120].

## Figures and Tables

**Table 1:** Cell signaling pathways altered in the pathogenesis of hepatocellular carcinoma. EGF: epidermal growth factor, EGF: epidermal growth factor receptor, mTOR: mammalian target of rapamycin, MAPK: mitogen-activated protein kinase, STAT: signal transducer and activator of transcription, VEGF: vascular endothelial growth factor.

Signaling Pathway	Role in Hepatocarcinogenesis	Reference
Wnt/β-catenin, Notch and hedgehog	differentiation and development	de La Coste et al. [50]
		Qi et al. [51]
		Patil et al. [52]
p53/p21 and RB1	genomic stability and cell cycle regulation	Naka et al. [53]
		Zondervan et al. [54]
EGF/EGFR, PI3K/AKT/mTOR and RAS/MAPK	cell proliferation and survival	Motoo et al. [55]
		Xie et al. [56]
		Ito et al. [57]
VEGF/VEGFR, PDGF/PDGFR and FGF/FGFR	angiogenesis	Miura et al. [58]
		Neaud et al. [59]
		Shimoyama et al. [60]
JAK/STAT	cytokine and growth factor signaling transduction	Feng et al. [61]

**Table 2:** Known in vitro experiments regarding the antitumor effects of capsaicin on HCC. AMPK: AMP-activated protein kinase, HepG2: human hepatoblastoma cell line, TRAIL: tumor necrosis factor-related apoptosis-inducing ligand.

Cell Line Information	Effects	Reference
**Sole treatment (capsaicin)**		
PLC/PRF/7, HuH7 and HepG2	Inhibition of proliferation	[69]
HepG2	Free fatty acids reduction	[72]
HepG2	Increased ROS production	[86,93,101,107]
HepG2	Induction of apoptosis	[71,86,93,96,101]
HepG2	Stimulation of autophagy	[93]
SK-Hep-1	Induction of apoptosis	[95]
Hep3B and HepG2	Facilitation of TRAIL-mediated apoptosis	[97]
**Co-treatment (capsaicin + sorafenib)**		
PLC/PRF/7, HuH7, and HepG2	Promotion of apoptosis	[69]
HepG2 and Huh-7	Increased apoptosis and induced AMPK activation	[81]
LM3, Hep3B, and HuH7	Tumor growth suppression	[114]
LM3	Stronger induction of apoptosis	[114]

**Table 3:** Known in vivo experiments regarding the antitumor effects of capsaicin on HCC. PPARδ: peroxisome proliferator-activated receptor gamma.

System Model	Effects	Reference
**Sole treatment (capsaicin)**		
Wild type mice	Enhancement of PPARδ and autophagy-related proteins	[72]
**Co-treatment (capsaicin + sorafenib)**		
Nude mice with PLC/PRF/5 xenografts	Tumor growth suppression	[69]
Athymic nude-Foxn1 mice injected with HepG2 or Huh-7 cells	Enhanced tumor growth reduction effect	[81]
BALB/C nude mice injected with LM3 cells	Suppression of cell growth, invasion and metastasis	[114]
